# A Day Seven Reflection Circle to Enhance Resilience in Residents after a Difficult Patient Loss

**DOI:** 10.15694/mep.2019.000058.1

**Published:** 2019-03-19

**Authors:** Nirvani Goolsarran, Aditi Bhagat, Stephen G Post

**Affiliations:** 1 Stony Brook University Hospital

**Keywords:** Resident well being, wellness, medical education, burnout, well-being

## Abstract

This article was migrated. The article was marked as recommended.

Caring for the dying patient can have significant impact on physicians in training, and if unaddressed, can lead to burnout and potentially compromised patient care. The literature suggests didactics and real time supportive interventions such as “post code debriefs” may be most effective in addressing the impact of death on physicians. In this paper, we highlight and discuss a reflection that is conducted several days after the event, when resident physicians are more self-aware of their mental hygiene and the residual impact of challenging event on their personal and professional well-being.

## Introduction

“I am unable to go on vacation because I am sad and torn about a recent patient death,” lamented a resident during routine patient care. Facing an unexpected mortality can be bewildering for trainees with little preparedness or coping skills. Processing such experiences in small reflection groups is important for resident resilience, well-being and professional identity development. Many residency programs have some structured well-being or wellness curricula in the form of didactics, and some may have an immediate post-resuscitation “debrief the grief” meeting that at least allows residents to minimally acknowledge the human side of such losses with a few minutes pause before rushing on to the next patient ( Eng et al. 2015). However, residents may carry unresolved emotional burdens from challenging losses that are hidden underneath the thin veneer of everyday equanimity. These burdens, including self-guilt for a serious medical error, can have adverse emotional consequences long term. Thus, there is a need for an on-demand substantive crisis intervention that we describe below as the Day Seven Reflection Circle (Day7RC).

Healthcare settings may very rarely utilize a formalized mechanism to address the psychological impact of traumatic events known as Critical Incident Stress Debriefing (CISD). CISD usually occurs immediately in the aftermath of a major disaster, and is designed to support overwhelmed disaster responders. They are heavily structured, time intensive, and are conducted by trained facilitators ( True 1999). Such a mechanism does not transpose to the resident in a clinical setting who is struggling to process a difficult clinical experience. We urge residency programs to develop their own unique intervention occurring about one week after such an experience occurs, especially to address “self-blame.” Therefore, we utilize what we now term the Day Seven Reflection Circle, a mechanism available as residents are more self-aware of their mental hygiene and the residual impact of challenging event on their personal and professional well-being. The seventh day is not designated based on scientific analysis about the optimal timing for such an intervention, but rather because it likely represents the temporal window when residents may have become clearly aware that they need an opportunity for emotional expression, discussion and guidance. Our anecdotal experience strongly supports this chronology.

## Methods

Despite holding well-being and resilience retreats, establishing a formalized wellness curriculum and conducting post resuscitation debriefs, we were surprised by our resident’s comment. His comment and feelings inspired us to start the Day7RC to enhance resident resilience, formation, and well-being. This is best described as a lightly facilitated opportunity for the residents to share their personal narrative about a critical event with several respected members of the healthcare team in confidentiality and trust. The Circle creates a safe forum for resident expression of thoughts and emotions, to normalize group member’s reactions to critical incidents, and to facilitate emotional recovery through perspective gaining and reframing. Additionally, it allows for follow up and suggestions for counseling as needed.

A week after the aforementioned patient death, an attending physician came to hear of the impact this adverse event had on the residents. The residents revealed their elevated self-awareness of how affected they really were. Therefore, a Circle session was convened with the three residents involved in the case and internal medicine attending physicians who serve as their mentors. The Circle session lasted for slightly more than one hour, allowing for meaningful and ample conversation.

Based on an evaluation of debriefing developed by Redinbaugh et al. (2003) at Memorial Sloan Kettering Cancer Center in a pilot study, the involved residents gave feedback on the Circle session. Unanimously, they found the Circle to be very helpful, and especially valued the opportunity to express how they felt with those who were also involved in the case and shared similar feelings. Residents noted that they came to better accept the fact that all physicians make mistakes for which self-forgiveness and self-compassion are needed, and that they were able to achieve these resilience assets through the attentive listening and renewed affirmation on the part of their mentors. Further, it was helpful for residents to hear attending physicians share narratives of their own experiences involving patient loss with self-doubt and resilience when they felt that their own performance was suboptimal and blameworthy. Residents came to understand that even the most experienced physicians have patients who have affected them and carry these emotions and lessons with them over the years. Residents reported poor coping skills before the session; all of them reported perseverating on the minutia of the case and feeling “incompetent.” They appreciated the invite to participate in the Circle as an opportunity to move forward with perspective and greater resilience. They reported having gained peace of mind and reassurance that they are still skilled resident physicians, and recommended the Circle for their co-residents as needed.

## Discussion

The Day7RC is *not* focused on the patient or the case, and is thus very different in content and tone from a Morbidity & Mortality session. Neither is it focused on an analysis of the case, although residents asked to go through the case analytically at a different time to learn from an academic standpoint. It is focused on the human side of the resident experience, examining feelings and enabling cognitive perspective and emotional resilience. Having a Day7RC in the hospital setting means that these feelings and responses are not left outside the building in the parking lot never to be resolved, or brought home to surprised spouses and friends who generally are unprepared to respond insightfully.

Ideally, a residency program might have a Circle of Trust series of debriefing processes to address the emotional reactions following each event ( Table 1), within which the Day7RC is embedded. We are convinced anecdotally that having such an interventional structure established within the culture of our residency can be protective and enhancing of professional identity formation and wellness.

**Table 1.** Outline of the Circle of Trust Chronology

Circle of Trust process	Time Frame process should occur
Immediate informal **“debrief the grief”**session right at the temporal node of loss simply to acknowledge the human side of the event	Within minutes of the event
A small **Day Seven Reflection Circle (Day7RC)** comprised of no more than five or six people including the resident(s) about one week after said event as requested by the resident	Seven days following the event
A one-on-one **“check in”** follow-up between the residency director/chief resident and the requesting resident within 14 to 21 days.	14-21 days after the event
**Schwartz Rounds ( Goodrich, 2012)**, which focuses on the human side of clinician experience with difficult clinical experiences and cases	Several months to a year from the event

## Take Home Messages

• Careful Debriefs interventions after patient death should not only focus on immediate debrief but should ideally entail some form of reflection a few days after the event to address emotional impact.
• Establishment of an interventional structure within the culture of our residency can be protective and enhancing of professional identity formation and wellness.
• Trainees found it helpful to hear attending physicians share narratives of their own experiences involving patient loss.

## Notes On Contributors

Dr. Nirvani Goolsarran (NG) is an Associate Professor of Clinical Medicine, Associate Program Director of Internal Medicine Residency, Director of Quality and Safety Education, Department of Medicine, Stony Brook University Hospital, Stony Brook, NY.

NG wrote the original draft of the paper.

Dr. Aditi Bhagat (AB) is a third year resident, Department of Medicine, Stony Brook University Hospital, Stony Brook, NY.

AB conducted the resident surveys, analyzed the data and added to the manuscript.

Dr. Stephen G. Post (SGP) is a Professor of Family, Population and Preventive Medicine, Director of the Center for Humanities, Compassionate Care and Bioethics, Stony Brook University Hospital, Stony Brook, NY.

SGP provided final revision and edits to the manuscript.
