# Supplementary material for: A Day Seven Reflection Circle to Enhance Resilience in Residents after a Difficult Patient Loss
Source: MedEdPublish (2016). 2019 Mar 19;8:58. [Version 1] doi: 10.15694/mep.2019.000058.1 (PMC10712625; doi:10.15694/mep.2019.000058.1)
Supplement: Peer review history

# Reviewer report: Reviewer response for version 1

**DOI:** 10.21956/mep.19784.r29738

**Published:** 2019-03-24

**Authors:** BALAJI ARUMUGAM

**Affiliations:** 1 TAGORE MEDICAL COLLEGE AND HOSPITAL

This review has been migrated. The reviewer awarded 4 stars out of 5 This is the very interesting and useful opinion on "The residents sharing their personal narrative about a critical event with several respected members of the healthcare team in confidentiality and trust. The circle session was well conducted I guess so that the physicians and the residents were freely exchanging their views on the seventh of the event. The physicians shared narratives of their own experiences involving patient loss and they felt that their own performance was suboptimal and blameworthy. This is the very important aspect wherein the residents should learn and to carry over message for their future practice. Similarly, the residents reported poor coping skills before the session; all of them reported perseverating on the minutia of the case and feeling “incompetent.” Such sort of the circle team can really help the physician and residents who deal with dying patients to cope up with the mental well being and can be a part of the circle team in future. I would suggest such long term programme involving the psychiatrist in the tertiary care institutes to maintain the confidentiality and the mental health of the intensive care team who mainly deal with these terminally ill patients and dying patients. This is the really a well written personal opinion on Day 7 Reflection circle and I felt the need to include such in the Hospital system.

Reviewer Expertise:

NA

No decision status is available

# Reviewer report: Reviewer response for version 1

**DOI:** 10.21956/mep.19784.r29739

**Published:** 2019-03-22

**Authors:** Gerard Flaherty

**Affiliations:** 1 National University of Ireland Galway

This review has been migrated. The reviewer awarded 3 stars out of 5 This is an interesting opinion piece which describes a single institution’s experience with a novel reflective exercise to support physician residents whose patients have died under their care. The article is well written with relevant supporting literature and a clear description of the format of the “day 7 reflection circle”, referred to in Table 1 as being part of a longitudinal “circle of trust”. The title suggests an intervention designed to enhance resident resilience but the article does not provide any objective measure of increased resilience. Qualitative feedback from participants is summarised, however, which points to the perceived effectiveness of the debriefing process. How would you evaluate its impact further? Is it envisaged that participation in this process would be voluntary or do the authors suggest that it should be a mandatory part of the debriefing procedures already in place? Why was a one week interval selected as the optimum time point for this intervention? Is this an arbitrary temporal window? Brief reference is made to “anecdotal experience” but this should be expanded upon. There is no demographic information provided on the participating residents – age, gender, residency grade, experience of bereavement, for example. Did the experienced physician facilitators receive any special training to enable them to conduct these sessions? You mention Schwartz Rounds in Table 1. How frequently are these held at your institution and what additional value is derived from an earlier Day7RC? Perhaps the authors could elaborate briefly on this. Overall, this is an interesting manuscript which addresses an important issue in medical professional training. It may be difficult for other institutions to replicate the intervention, however, without providing some additional detail as suggested in this review.

Reviewer Expertise:

NA

No decision status is available

# Reviewer report: Reviewer response for version 1

**DOI:** 10.21956/mep.19784.r29736

**Published:** 2019-03-19

**Authors:** Johnny Lyon Maris

**Affiliations:** 1 Southampton GP Education Unit

This review has been migrated. The reviewer awarded 3 stars out of 5 Thankyou for this descriptive intervention. It is a topical issue and one close to many physician's hearts. The description of the circle is clear, the text well written and the purpose very necessary. It would be good to see if your institution can sustain this intervention as an hour of 5-6 clinicians time, although for a very valuable reason, is increasingly scarce. It would be good to hear who decides that a death is a 'Difficult Patient Loss', with resource ramifications. It would also be useful to hear what the evaluation results are from all the participants - you suggest the more experienced clinicians would be helping the less experienced. It is my personal feeling that the support can be mutually supportive rather than one-way.Thankyou for opening up the discussion and I look forward to hearing more about the practicalities and evaluation.

Reviewer Expertise:

NA

No decision status is available

# Reviewer report: Reviewer response for version 1

**DOI:** 10.21956/mep.19784.r29737

**Published:** 2019-03-19

**Authors:** Susmita Reddy Karri

**Affiliations:** 1 Topiwala national medical college and BYL nair charitable hospital, mumbai. india

This review has been migrated. The reviewer awarded 3 stars out of 5 Touched upon an important topic that every physician encounters at some point in his/her career. I personally believe that the new/ junior doctors are more affected by this. While I agree that the senior physicians can help the juniors on how to deal with it, I do think that we should look at having a small team/committee in every hospital where doctors can go and share their grief.

Reviewer Expertise:

NA

No decision status is available
